# Metabolic and Molecular Subacute Effects of a Single Moderate-Intensity Exercise Bout, Performed in the Fasted State, in Obese Male Rats

**DOI:** 10.3390/ijerph18147543

**Published:** 2021-07-15

**Authors:** Éverton Lopes Vogt, Maiza Cristina Von Dentz, Débora Santos Rocha, Jorge Felipe Argenta Model, Lucas Stahlhöfer Kowalewski, Samir Khal de Souza, Vitória de Oliveira Girelli, Paulo Ivo Homem de Bittencourt, Rogério Friedman, Mauricio Krause, Anapaula Sommer Vinagre

**Affiliations:** 1 Department of Physiology, Universidade Federal do Rio Grande do Sul (UFRGS), Porto Alegre 90050-170, RS, Brazil; evvogt@gmail.com (É.L.V.); maizavondentz@hotmail.com (M.C.V.D.); debora.santosrocha@gmail.com (D.S.R.); jorgefamodel@gmail.com (J.F.A.M.); samirks@hotmail.com (S.K.d.S.); vvitoriagirelli@gmail.com (V.d.O.G.); anapaula.vinagre@ufrgs.br (A.S.V.); 2 Laboratory of Inflammation, Metabolism and Exercise Research (LAPIMEX) and Laboratory of Cellular Physiology, Department of Physiology, Institute of Basic Health Sciences, Universidade Federal do Rio Grande do Sul, Porto Alegre 90050-170, RS, Brazil; lucaskowalewski7@gmail.com (L.S.K.); pauloivo@ufrgs.br (P.I.H.d.B.J.); 3 Endocrine and Metabolic Unit, Hospital de Clinicas de Porto Alegre, Universidade Federal do Rio Grande do Sul, Porto Alegre 90040-060, RS, Brazil; rogeriofriedman@gmail.com; 4 Graduate Program in Medical Sciences: Endocrinology, Department of Internal Medicine, Faculty of Medicine, Universidade Federal do Rio Grande do Sul, Porto Alegre 90035-903, RS, Brazil

**Keywords:** fasting, aerobic exercise, obesity, metabolism, inflammation

## Abstract

*Introduction and objectives*: Obesity represents a major global public health problem. Its etiology is multifactorial and includes poor dietary habits, such as hypercaloric and hyperlipidic diets (HFDs), physical inactivity, and genetic factors. Regular exercise is, per se, a tool for the treatment and prevention of obesity, and recent studies suggest that the beneficial effects of exercise can be potentiated by the fasting state, thus potentially promoting additional effects. Despite the significant number of studies showing results that corroborate such hypothesis, very few have evaluated the effects of fasted-state exercise in overweight/obese populations. Therefore, the aim of this study was to evaluate the subacute effects (12 h after conclusion) of a single moderate-intensity exercise bout, performed in either a fed or an 8 h fasted state, on serum profile, substrate-content and heat shock pathway–related muscle protein immunocontent in obese male rats. *Methods*: Male Wistar rats received a modified high-fat diet for 12 weeks to induce obesity and insulin resistance. The animals were allocated to four groups: fed rest (FER), fed exercise (FEE), fasted rest (FAR) and fasted exercise (FAE). The exercise protocol was a 30 min session on a treadmill, with an intensity of 60% of VO_2_max. The duration of the fasting period was 8 h prior to the exercise session. After a 12 h recovery, the animals were killed and metabolic parameters of blood, liver, heart, gastrocnemius and soleus muscles were evaluated, as well as SIRT1 and HSP70 immunocontent in the muscles. *Results*: HFD induced obesity and insulin resistance. Soleus glycogen concentration decreased in the fasted groups and hepatic glycogen decreased in the fed exercise group. The combination of exercise and fasting promoted a decreased concentration of serum total cholesterol and triglycerides. In the heart, combination fasting plus exercise was able to decrease triglycerides to control levels. In the soleus muscle, both fasting and fasting plus exercise were able to decrease triglyceride concentrations. In addition, heat shock protein 70 and sirtuin 1 immunocontent increased after exercise in the gastrocnemius and soleus muscles. *Conclusions*: An acute bout of moderate intensity aerobic exercise, when realized in fasting, may induce, in obese rats with metabolic dysfunctions, beneficial adaptations to their health, such as better biochemical and molecular adaptations that last for at least 12 h. Considering the fact that overweight/obese populations present an increased risk of cardiovascular events/diseases, significant reductions in such plasma markers of lipid metabolism are an important achievement for these populations.

## 1. Introduction

Resulting from an imbalance between acquired and expended calories, overweight and obesity have replaced tobacco consumption as the number one lifestyle-related major health problem worldwide [1,2,3]. According to the World Health Organization (WHO), overweight and obesity are the fifth highest risk factor for global deaths [4]. At least 2.8 million adults die each year because of being overweight or obese. Dietary fat plays a major role in obesity and is linked with the development of several chronic health problems, such as hyperglycemia, dyslipidemia, hypertension, type 2 diabetes mellitus (T2DM) and certain types of cancer [3,5].

The low-grade inflammation induced by adipose tissue expansion is a hallmark of obesity and metabolic diseases [6], leading to the chronic release of cytokines, such as TNFα, IL-6 and IL-1β, which leads to the activation of serine threonine kinases such as c-Jun N-terminal (JNK) and IκB kinase kinase (IKK) [7]. As a result, both JNK and IKK phosphorylate insulin receptor (IR) substrate-1 (IRS-1) on Ser-307, leading to the inactivation of the insulin receptor downstream response [7] and thus causing insulin resistance. Lipid oversupply and hyperglycemia can lead to increased deposition of lipid species such as diacylglycerols and ceramides, which can also activate JNK and IKK in liver and/or skeletal muscle, leading to insulin resistance and sustained hyperglycemia and hyperlipemia [8]. In addition to cytokines, the chronic presence of saturated fatty acids in the diet can directly induce chronic inflammation and its complications [9,10,11]. This inflammatory effect can be partially mediated through their binding to Toll-like receptors (TLRs), particularly 2 and 4, inducing the activation of pro-inflammatory pathways that can activate JNK and protein kinase C (PKC), both inhibitors of the insulin signaling, thus promoting insulin resistance and cellular dysfunction in several tissues, such as smooth muscle cells, cardiac tissue, skeletal muscle, hepatocytes, endothelial cells and others [12,13,14].

The economic and social consequences of obesity and inflammation-associated diseases represent a challenge in terms of assuring sufficient resources and effective health services. Therefore, strategies to prevent and treat obesity are essential, involving a series of lifestyle modifications that include diet and exercise interventions, pharmacological therapy and bariatric surgery [15,16,17]. Physical exercise, an important component of obesity treatment and prevention, can alter the biochemical and molecular machinery to modify the mobilization of energetic substrates [17,18]. Recent studies suggest that the beneficial effects of exercise can be potentiated if performed while fasting, thus promoting additional effects in comparison to the fed state, such as higher lipid mobilization and oxidation as well as superior metabolic adaptations [19].

Fasting is characterized by the absence of food intake for a period of time that lasts from several hours to a few weeks [20], resulting in a metabolic switch in the use of energy substrates in which non-esterified fatty acids (NEFAs), ketone bodies and glucose derived from liver glycogen and gluconeogenesis are the predominant energy sources for ATP production [21]. Fasting is capable of activating a series of transcription factors responsible for stimulating the transcription of enzyme genes with a crucial role in metabolic pathways, such as lipoprotein lipase (LPL), muscle carnitine palmitoyltransferase I (CPT I) and long-chain acyl-CoA dehydrogenase (LCAD), changing the rate of gluconeogenesis, fatty acid oxidation and ketogenesis [22]. Similarly to fasting, aerobic exercise, particularly that of low-to-moderate intensity, induces an increased lipolytic activity that results in higher plasma NEFA availability, causing an important metabolic shift that leads to the increased utilization/oxidation of fat rather than carbohydrates [23]. In fact, conditions that lead to increased supply of free fatty acids to skeletal or cardiac muscle reduce utilization and oxidation of glucose [24]. In both conditions (fasting and exercise), these changes are attributed to several hormonal modifications, including increased adrenaline and decreased insulin secretion in the blood [25].

In addition to the metabolic changes, both fasting and exercise can induce positive adaptations in terms of inflammatory control. Resolution of inflammation is key to improving insulin sensitivity and metabolism [26]. The heat shock response (HSR) is one of the molecular pathways capable of promoting insulin sensitivity and decreasing inflammation [27]. The nicotinamide adenine dinucleotide (NAD^+^) dependent deacetylase sirtuin 1 (SIRT1) is activated by the increase in the NAD^+^/NADH ratio, induced by metabolic stress/demand (fasting and exercise) [28]. SIRT1 prolongs the heat shock factor 1 (HSF1) binding to the promoter regions of heat shock genes by maintaining HSF1 in a deacetylated and DNA-binding competent state, enhancing the transcription of molecular chaperones such as heat shock proteins 70 (HSP70) and 25 (HSP25) [29,30]. HSP70 is a classical molecular chaperone that interacts with other proteins (unfolded, in non-native state and/or stress-denatured conformations), avoiding inappropriate interactions, formation of protein aggregates and degradation of damaged proteins as well as helping the correct refolding of nascent proteins [31]. In addition to its several functions (anti-apoptosis, protein translocation, metabolism and others) [27], this protein exerts, intracellularly, a potent anti-inflammatory effect [32]. The anti-inflammatory effect of HSP70 is attributed mainly to its interaction with NF-κB, which is increased together with IKK in different obese experimental models [27]. The role of HSR in insulin sensitivity has been recently demonstrated, since HSP70 was shown to protect against high-fat-diet- and obesity-induced hyperglycemia, hyperinsulinemia, glucose intolerance and insulin resistance [7,33,34].

Recent evidence has shown that, when compared with exercise performed in the fed state, fasting aerobic exercise increases the fat contribution as an energy substrate after exercise for up to 24 h [35,36,37,38,39,40]. Some authors [41,42] claim that low-to-moderate intensity aerobic exercise performed on an empty stomach offers no advantage with regard to fatty acid oxidation when compared to exercise performed in a fed state, while others [23,43] show that, in skeletal muscle, exercising while fasting stimulates the use of fatty acids as the primary source of energy while suppressing glucose metabolism, as compared to the fed state. However, it is worth mentioning that these studies evaluated only healthy and lean individuals. Since higher fat oxidation capacity during exercise seems to be related to a decrease in the number of metabolic risk factors, aerobic exercise performed in the fasted state could be considered a strategy to increase fat oxidation and to promote adaptations that may be beneficial to health, particularly with regard to obesity [44]. Therefore, it can be hypothesized that the use of simultaneous metabolic challenges (exercise and fasting) would induce several changes in skeletal muscle cellular signaling and in lipid, protein, and carbohydrate metabolism.

Despite the significant number of studies showing results that corroborate such hypotheses, only a few research studies have evaluated the effects of exercise in the fasted state in overweight/obese populations. Therefore, the aim of this study was to evaluate the subacute effects (12 h after its execution) of a single moderate-intensity exercise bout, performed in the fed or fasted state, on biochemical and molecular parameters in HFD obese male Wistar rats.

## 2. Materials and Methods

### 2.1. Animals and Ethics

Male Wistar rats Rattus novergicus (*n* = 30), 60 days old, were obtained from Centro de Reprodução e Experimentação de Animais de Laboratório (CREAL) of Universidade Federal do Rio Grande do Sul (UFRGS). The animals were maintained in standard bioterium conditions: 12 h light/dark cycle, controlled temperature (21 ± 2 °C), 70% relative humidity, with food and water ad libitum. This project was approved by the Ethics Committee on the Use of Animals (CEUA) of UFRGS (protocol 34271).

### 2.2. High-Fat Diet and Experimental Groups

The animals received a modified high-fat diet (HFD: 31.07% lipids, 49.09% carbohydrates and 8.18% proteins) for 12 weeks to induce obesity. Body mass was recorded weekly. To access glucose homeostasis and to test the efficiency of the HFD to induce insulin resistance, an oral glucose tolerance test (OGTT) and an injected (intraperitoneally) glucose tolerance test (ipGTT) were performed at the end of the treatment, and the results were compared with an additional control group (fed a standard diet). This lean control animals were used only in this test because we were interested in evaluating the effects of fasting and exercise in individuals with diet-induced obesity. In the last week of treatment, all animals performed one week of acclimatization to the treadmill, which consisted of five daily 15 min sessions at very low speed and 0° treadmill grade. A washout period of 5 days, between the last day of acclimatization and the acute exercise session, was included to avoid any adaptations or subacute effects of the last acclimation session. At the end of the experimental treatment, the animals were randomly allocated to one of the four experimental groups: fed rest (FER), fed exercise (FEE), fasted rest (FAR) and fasted exercise (FAE).

### 2.3. Glucose Tolerance Tests

Glucose was given by gavage (OGTT) (1 g/kg) or injected intraperitoneally (IPGTT) (1 g/kg) to overnight fasted mice, and glycemia was measured using a glucometer (On Call Plus, Acon Laboratories Inc., San Diego, CA, USA) at 0, 30, 60, 90 and 120 min after glucose administration. For both tests, the area under the curve (AUC) was calculated.

### 2.4. Exercise Protocol and Experimental Design

**Figure 1:**
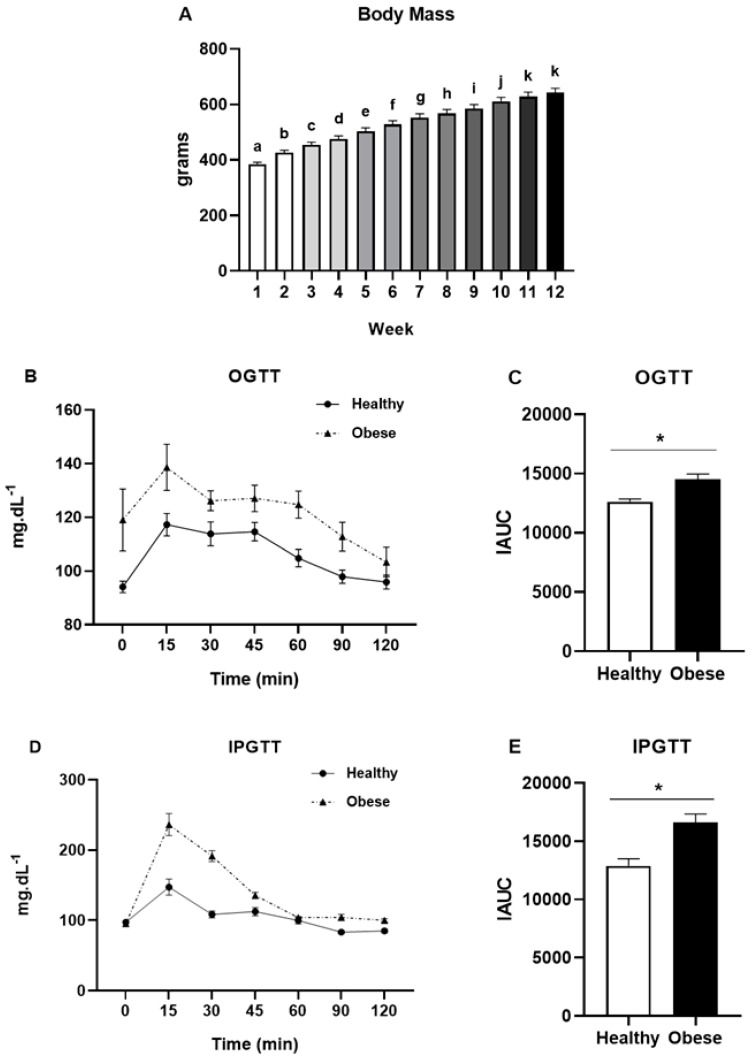
Total body mass (**A**) of the rats during the treatment with high-fat diet. Oral glucose tolerance test (OGTT) (**B**) and OGTT incremental area under the curve (IAUC) (**C**) on the 11th week of treatment. Intraperitoneal glucose tolerance test (IPGTT) (**D**) and IPGTT incremental area under the curve (IAUC) (**E**) on the 11th week of treatment. Data were analyzed by two-way ANOVA (Bonferroni post hoc), one-way ANOVA (Bonferroni post hoc) or Student’s *t* test. Results were considered different when *p* < 0.05. Letters represent significant differences between weeks. * significant difference between experimental groups.

Prior to the exercise session, animals from the FAR and FAE groups were subjected to a fasting period of 8 h [45]. The acute exercise consisted of a 30 min session on the treadmill, with speed and inclination corresponding to an intensity of 60% of VO_2_max (10 m/min, 0° treadmill grade), according to Rodrigues et al. [24]. Since the VO_2_ was not directly tested, we measured the lactate concentration before and after the exercise. Confirming that our exercise protocol was aerobic (moderate), no changes in lactate were found between rest and immediately post-exercise (Figure 1, Supplemental Material). To test the subacute effects of our intervention, the animals were euthanized 12 h after the exercise session. Control animals were kept in rest and killed at the same time. During this recovery period, the animals were resubmitted to their high-fat diet. The animals were killed by decapitation, and the blood, liver, heart, soleus, and gastrocnemius samples were collected. Blood samples were centrifuged (10 min, 1510 g), and the serum was used to evaluate glucose, total proteins, total cholesterol, and triglyceride (TGL) levels. Glycogen, lactate and triglyceride concentrations were determined for the tissue samples. HSP70 and sirtuin 1 immunocontent were determined for skeletal muscle samples. From the 32 animals, two refused to perform the exercise bout (1 from each exercise group). For this reason, they were excluded from the analysis.

### 2.5. Biochemical Analyses

Glucose, lactate, total proteins, total cholesterol, and triglycerides in the serum were measured using enzymatic assay kits (Labtest Diagnóstica SA, Lagoa Santa, Minas Gerais, Brazil) for spectrophotometer analysis. The concentrations of glucose, triglycerides, lactate, and cholesterol were expressed as mg·dL^−1^ of plasma and total proteins as g·dL^−1^. The concentration of glycogen in the tissue samples was determined as previously described [46]. Briefly, the tissue samples were first homogenized with 30% KOH, then washed with ethanol, hydrolyzed with 4N HCl, neutralized with 2 M Na_2_CO_3_ and finally diluted with water. The levels of glycogen were determined as glucose equivalents using a glucose kit and expressed as mg glycogen.g tissue^−1^ (wet weight). To determine lactate concentration, tissue samples were homogenized with 0.9% saline solution in tubes previously treated with 0.1 M NaF. Lactate was measured using the same assay kit as used for plasma. Results were expressed as mg lactate.g muscle^−1^ [47]. To extract triglycerides, tissue samples were homogenized with 0.9% saline in a ratio of 10:1 (1 mg of tissue to 10 μL of saline), and the concentration of triglycerides was determined with the same kit as used for the plasma assay [46]. The results were expressed as mg·g^−1^ (wet weight).

### 2.6. SDS-PAGE and Immunoblotting Analysis

For protein separation, SDS-PAGE (polyacrylamide gel electrophoresis with sodium dodecyl sulfate, mini-PROTEAN^®^ 3 Electophoresis Cell, BioRad, Hercules, CA, USA) was used with a polyacrylamide concentration of 10%. Approximately 30 µg of the protein extracted from the samples was incubated with Laemmli solution and added to each well of the gel for electrophoresis. After the electrophoresis, the polyacrylamide gel was removed from the glass plates and placed in the electrotransfer module in contact with the nitrocellulose (NC) membrane and covered with transfer buffer. The transfer was made in the Trans-Blot SD system (Semi Dry Electrophoretic Transfer Cell, BioRad, USA) for one hour, at a potential difference of 25 V. The NC membranes containing the proteins were then incubated for one hour in blocking solution. After blocking, the NC membranes were incubated with antibodies (catalog numbers SAB4200714: HSP70; AV32386: SIRT1; Sigma Aldrich, Saint Louis, MO, USA) and diluted in Tween Tris Buffer Saline (TTBS) for at least sixteen hours at 4 °C, under constant agitation. After incubation, the membranes were washed with TTBS (1%) and then incubated with the second antibody (catalog number A9044: Anti-Mouse IgG (whole molecule)—Peroxidase antibody produced in rabbit—IgG fraction of antiserum, buffered aqueous solution, Sigma Aldrich) for two hours at room temperature. Soon after, the membranes were washed with Tris Buffer Saline (TBS) and incubated in a dark room with chemiluminescence solution for one minute. The chemiluminescence reaction was performed using a detection system based on luminescent substrates (luminol and p-cumaric acid). After incubation with the chemiluminescence solution, the NC membranes were placed in contact with a photographic film (GE Healthcare^®^/Amersham HypperfilmTM ECL, Darmstadt, Germany). After developing, the film was analyzed by optical densitometry, and the bands were measured by image processing (ImageMaster VDS, Pharmacia Biotech, San Diego, CA, EUA). The results were expressed in arbitrary units (AUs).

### 2.7. Statistical Analysis

The oral (OGTT) and injected (ipGTT) glucose tolerance tests of lean and HFD obese animals were analyzed by the area under the curve (AUC). The results obtained in the diet-induced obese animals submitted to fasting and exercise were first analyzed with the Kolmogorov-Smirnov normality test to check the distribution of the data and, subsequently, by the Levene’s homogeneity test. Normally distributed data were analyzed using two-way ANOVA to compare the effects of fasting (fed × fasting) and exercise (rest × exercise), as well as the interaction between these factors, with Bonferroni’s post hoc for homogeneous data or Games-Howell for non-homogeneous data. Non-parametric data were examined with the Kruskal-Wallis non-parametric test, complemented by Dunn’s post-test. Results were considered statistically different when *p* < 0.05. Analyses were performed with the Statistical Package for Social Sciences (SPSS version 25.0, IBM, Armonk, NY, USA).

## 3. Results

The animals’ total body mass increased progressively (F(2.34,72.62) = 317.212; *p* < 0.01) along the treatment (Figure 1A). A high-fat diet, when compared to a usual diet, increased insulin resistance, as depicted in Figure 1B–D.

**Figure 2:**
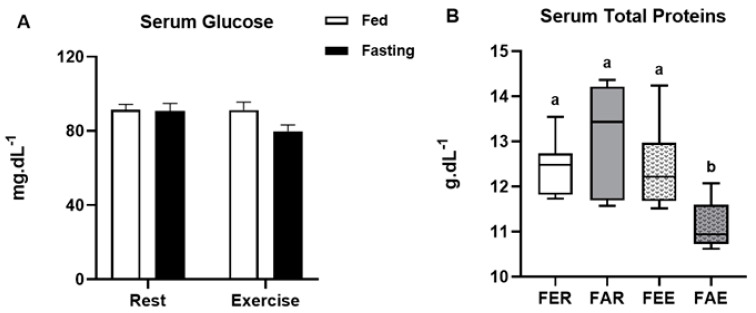
Concentration of glucose (**A**) and total proteins (**B**) in the serum at the end of the experimental treatment. Animal groups: fed rest (FER), fed exercise (FEE), fasted rest (FAR) and fasted exercise (FAE). Data were analyzed by two-way ANOVA (Bonferroni post hoc) or Kruskal-Wallis test. Results were considered different when *p* < 0.05. Letters represent significant differences between each experimental group.

**Figure 3:**
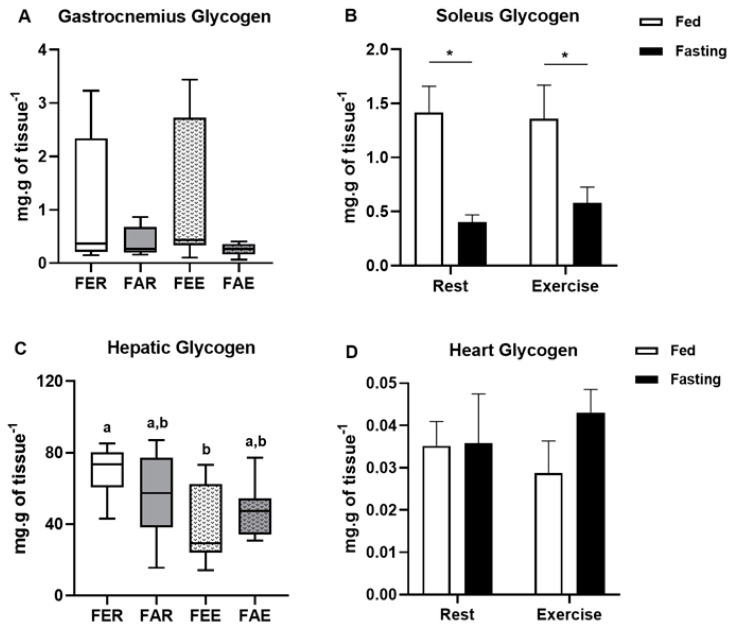
Glycogen concentration in the gastrocnemius (**A**), soleus (**B**), liver (**C**) and heart (**D**) of the rats at the end of the experimental treatment. Animal groups: fed rest (FER), fed exercise (FEE), fasted rest (FAR) and fasted exercise (FAE). Data were analyzed by two-way ANOVA (Bonferroni post hoc) or Kruskal-Wallis test. Results were considered different when *p* < 0.05. * significant difference between fed and fasted groups. Letters represent significant differences between each experimental group.

**Figure 4:**
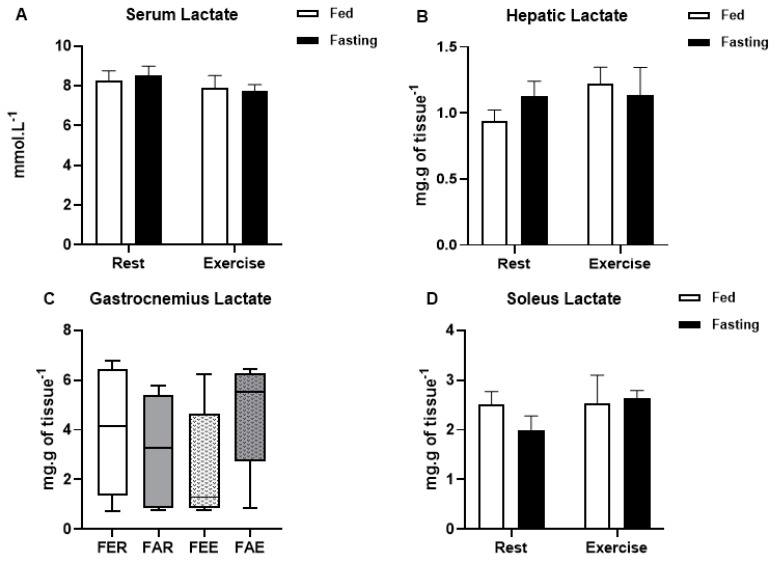
Lactate concentration in the serum (**A**), liver (**B**), gastrocnemius (**C**) and soleus (**D**) muscles at the end of the experimental treatment. Animal groups: fed rest (FER), fed exercise (FEE), fasted rest (FAR) and fasted exercise (FAE).

Blood glucose levels were not affected by fasting or by exercise, while total protein levels decreased only in the group submitted to fasting and exercise (x^2^(3) = 12.075; *p* < 0.01) when compared to the other groups (Figure 2A,B, respectively). Gastrocnemius glycogen concentration (Figure 3A) was not affected by fasting or exercise, while soleus glycogen concentration decreased in the fasted groups (2-way ANOVA, F(1,24) = 18.310, *p* < 0.01) (Figure 3B). Hepatic glycogen decreased in the fed exercise group when compared to the rest group (x^2^(3) = 9.294; *p* = 0.03) (Figure 3C). There were no differences in the glycogen content in the heart (Figure 3D). Lactate concentrations were not different between the groups in any of the analyzed samples, including serum, liver, gastrocnemius, and soleus muscles (Figure 4A–D).

**Figure 5:**
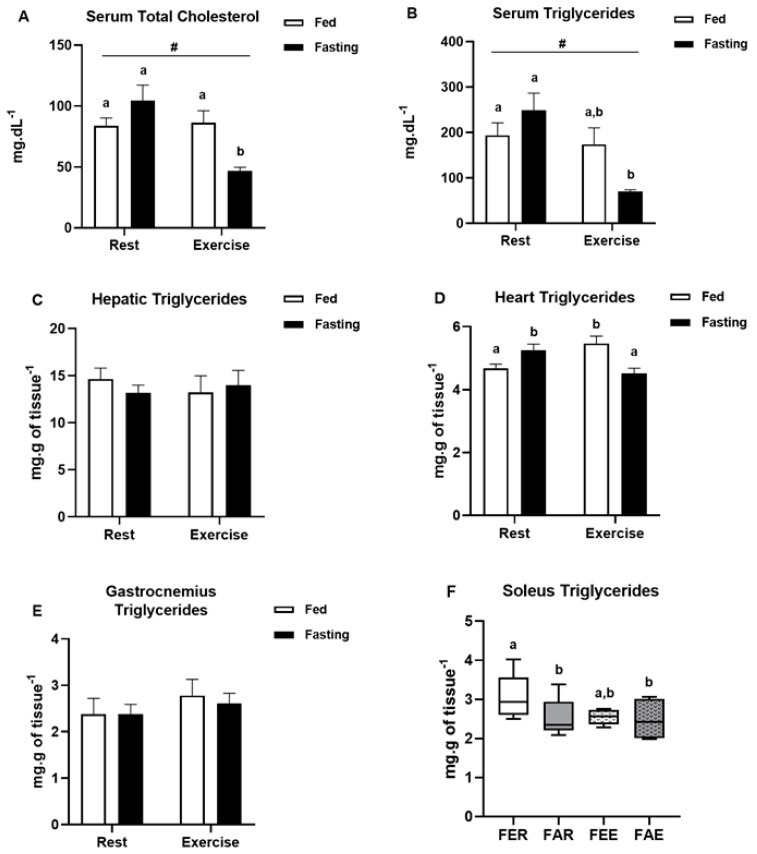
Concentration of lipids in the tissues. Serum total cholesterol (**A**). Triglycerides in the serum (**B**), liver (**C**), heart (**D**), gastrocnemius (**E**) and soleus (**F**) muscles at the end of the experimental treatment. Animal groups: fed rest (FER), fed exercise (FEE), fasted rest (FAR) and fasted exercise (FAE). Data were analyzed by two-way ANOVA (Bonferroni post hoc) or Kruskal-Wallis test. Results were considered different when *p* < 0.05. # Represents significant difference between rest and exercised groups. Letters represent significant differences between each experimental group.

Although fasting alone was not able to alter the concentrations of serum total cholesterol, there was a significant decrease caused by exercise (2-way ANOVA, F(1,25) = 9.168, *p* = 0.01) and by the interaction between exercise and fasting (2-way ANOVA, F(1,25) = 10.991, *p* < 0.01) (Figure 5A). Although fasting did not change serum triglycerides, both exercise (2-way ANOVA, F(1,25) = 10.799, *p* < 0.01) and the interaction between exercise and fasting (2-way ANOVA, F(1,25) = 6.894, *p* = 0.02) were able to significantly decrease these concentrations (Figure 5B). Liver triglyceride concentration did not change between the treatments (Figure 5C). In the heart, there was an interaction between fasting and exercise, and the combination of fasting plus exercise was able to decrease triglycerides to control levels (2-way ANOVA, F(1,25) = 17.169, *p* < 0.01) (Figure 5D). In the gastrocnemius muscle, the triglyceride concentration did not change between treatments, while in the soleus muscle, both fasting and fasting plus exercise were able to decrease triglyceride concentrations compared to the control group (x^2^(3) = 8.109; *p* = 0.04) (Figure 5E,F).

**Figure 6:**
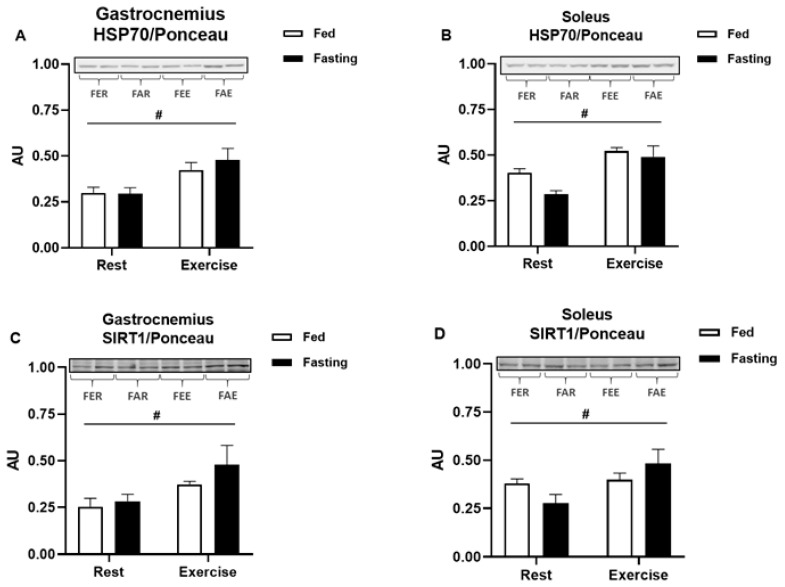
Protein immunocontent at the end of the experimental treatment. Heat shock protein 70 (HSP70) in the gastrocnemius (**A**) and soleus (**B**) muscles. Sirtuin 1 (SIRT1) in the gastrocnemius (**C**) and soleus (**D**) muscles. Data were analyzed by two-way ANOVA followed by Bonferroni post hoc. Results were considered different when *p* < 0.05. # Represents significant difference between rest and exercised groups.

Heat shock protein 70 (HSP70) immunocontent increased after exercise in the gastrocnemius (2-way ANOVA, F(1,26) = 13.301, *p* < 0.01) and soleus muscles (2-way ANOVA, F(1,24) = 19.861, *p* < 0.01) (Figure 6A,B). In both muscles, fasting was not able to modify HSP70 content, although exercise was able to increase it. Sirtuin 1 (SIRT1) deacetylase immunocontent behaved similarly to HSP70 in the gastrocnemius (2-way ANOVA, F(1,26) = 7.331, *p* = 0.01) and soleus (2-way ANOVA, F(1,26) = 6.020, *p* = 0.02) muscles (Figure 6C,D, respectively). Again, fasting did not change the protein content in the muscles, but exercise increased it significantly. Once again, there was no interaction between exercise and fasting.

## 4. Discussion

Exercise performed in a fasted state induced healthy metabolic effects on carbohydrate and lipid metabolism, such as reduced levels of serum cholesterol and triglycerides in the serum, heart, and soleus muscle, which lasted for up to 12 h after the exercise bout, in an obese-induced insulin-resistant rat model. The benefits of the combination fasting plus exercise were previously assessed in other animal and human studies [19]. However, different from our work, most studies investigated the effects of fasting and exercise in healthy and physically active individuals/animals, which were evaluated immediately after the exercise (i.e., acute effects, for up to 3 h). To our knowledge, this is the first study looking at subacute effects of exercise in the fasted state (12 h after the exercise intervention) in HFD obese and insulin-resistant rats.

Considering that the soleus and gastrocnemius exhibit different metabolic profiles, we evaluated triglycerides and glycogen in both muscles. Like previous studies [48,49], a decreased glycogen content was found in the skeletal muscles from fasted animals (statistically significant only in the soleus), with no differences between non-exercised and exercised animals. The same scenario occurred in the levels of triglycerides. This is an intriguing finding, since these animals were fed an HFD during the recovery period, prior to euthanasia (12 h). The inability to restore muscle glycogen levels during the fed state may be explained by the increased insulin-resistance state promoted by the HFD, which can be caused by low-grade inflammation, accumulation of intramuscular lipid metabolites, fatty-acid-induced toll-like receptor (TLR) activation and the higher rates of fatty acid uptake [6]. In fact, other models of obese insulin-resistant rats confirm that skeletal muscle glycogen remains reduced during the recovery period [50]. Acute exercise is known to improve glucose uptake and glycogen synthesis, even in insulin-resistant individuals [51]. However, this was not the case for our intervention, since exercised animals had low levels of muscle glycogen, even after a 12 h recovery period with free access to food. The increased lipolytic rate induced by fasting and exercise, along with increased fatty acid uptake and use in the exercise group (where an enhanced rate of beta-oxidation is expected) may have led to reduced glucose uptake (Randle’s cycle, [24]), reducing muscle capacity to use and store glucose as glycogen [24]. In addition, during the recovery time, the animals returned to their HFD, increasing the offer of lipids to the muscles, and resulting in diminished glucose metabolism. This increased fat metabolism (increased lipolytic activity, fatty acid uptake and use), may be responsible for the lower serum triglycerides and total cholesterol found in fasted exercised animals (Figure 5). However, we cannot exclude that our intervention changed appetite and food intake in the subsequent hours following exercise, thus inducing modifications in plasma metabolite concentration [52].

On the other hand, hepatic glycogen followed a different pattern, in which glycogen levels of fed and rested animals reduced 12 h after the time in which the other groups exercised (Figure 3C). This indicates that fed animals rely more on glucose metabolism during exercise, and therefore liver glycogenolysis is increased to maintain their glycaemia. In contrast, the animals that exercised during fasting were able to maintain their liver glycogen levels, probably because the increased availability of fatty acids induced a glycogen sparing effect. Higher rates of lipolysis during exercise in the fasted state (thus a greater release of glycerol) may result in increased gluconeogenesis during the recovery period (by using glycerol as a source to synthesize new glucose). In addition to the use of glycerol, amino acids and proteins could also be used for this purpose. This may explain why only this group of animals was able to recover their levels of glycogen in the hours after exercise. Apparently, the unchanged levels of lactate in all tissues indicates that this metabolite did not play an important role during the experimental intervention and confirms that the level of exercise was not intense.

Curiously, the content of total serum proteins was lower in the fasted-exercised group (Figure 2B). It is expected the fasting induces reductions in plasma protein concentration. However, this response usually occurs after at least 48 h of fasting [53,54]. Since all analyses were conducted 12 h after the intervention, and the animals were resubmitted to their high-fat diet during the recovery period, we suggest that exercise performed in the fasted state may accelerate the effect of fasting on plasma proteins. This effect may need to be considered for longer interventions (exercise training in fasted state), since lower levels of plasma protein can induce changes in oncotic pressure and thus water homeostasis (osmolality/volume/pressure).

In obese male rats that were submitted to exercise (in the fasted or fed state), SIRT1 and HSP70 expression remained increased in both studied skeletal muscles (gastrocnemius and soleus, Figure 6) for up to 12 h after the exercise bout. However, no additional effects of fasting were seen at 12 h after exercise. The increased HSP70 expression induced by exercise is important not only for metabolism but also for its implication in proteostasis and resolution of inflammation, particularly in a model of low-grade inflammation, such as the one in this study.

The metabolic switch that occurs in the fasting state and exercise depends on the activation of several proteins and transcription factors, considered as “master regulators” of metabolism, such as 5′ AMP-activated protein kinase (AMPK), SIRT1 and peroxisome proliferator-activated receptor gamma coactivator 1-alpha (PGC-1α) ([24]. The activation of these factors depends on the energetic status of the cells; thus, changing the levels of AMP/ATP and NAD+/NADH will lead to metabolic changes favoring catabolism and oxidative metabolism. In fact, endurance exercise in the fasted state results in the activation of AMPK and acetyl-CoA carboxylase-α [55]. Other molecular pathways that are sensitive to the changes in cellular energetic status that occur during metabolic challenges are NAD+-dependent deacetylase sirtuin 1 (SIRT1) and heat shock response (HSR) [56]. SIRT1 is a deacetylase that is activated by the increase in the NAD+/NADH ratio, induced by metabolic stress/demand. Interestingly, a direct crosstalk between SIRT1, AMPK, PGC-1α and HSR occurs in different cells [28]. For instance, SIRT1 can activate AMPK by deacetylation, inducing direct metabolic effects [57].

Other studies have shown that exercise is able to increase the expression and synthesis of HSP70 [33,58,59]. Its decreased intracellular concentration is correlated with insulin resistance [7,60], while the induction of its expression, by whatever route, is related to cell protection against inflammatory damage [61,62]. The anti-inflammatory role is due to its ability to inhibit the translocation of factors such as NF-kB (Nuclear Factor-kappa B) to the cell nucleus [32]; NF-kB activity is increased in obese individuals [60]. An increase in the intracellular HSP70 immunocontent is also able to oppose the phosphorylation and activation of pro-inflammatory proteins such as JNK (c-Jun N-terminal Kinase), induced by hyperlipidic diets [7], thus reducing its associated harmful effects.

During fasting, proteins such as SIRT1 are activated in the metabolic management of glycogenolysis for ketogenesis, which leads to the activation of HSF1 (Heat Shock Factor 1), a HSP70 transcription factor [32], the deacetylation and degradation of CRTC2 (CREB Regulated Transcription Coactivator 2), the main mediator of hepatic gluconeogenesis [63,64], and to indirect increase in fatty acid oxidation through the deacetylation of factors such as PGC1α (Peroxisome Proliferator-Activated Receptor Gamma Coactivator 1-α) [65,66] and activation of PPARα [67]. Thus, the effects of both exercise and fasting seem to contribute to a better overall metabolic profile, suggesting that their association can potentiate their effects, which would lead to a faster improvement in insulin resistance and low-grade chronic inflammation.

Most of the significant changes found in our intervention were induced when exercise was performed in the fasted state. The combination of these two metabolic challenges resulted in acute and subacute metabolic changes (particularly in fat metabolism) and in skeletal muscle signaling. Previous studies have shown that, despite the performance of fasting, physical training does not increase the content of FATCD36 (Fatty Acid Translocase) and UCP3 (Uncoupling Protein 3) in muscle tissue; the content of FABPpm (membrane-associated Fatty Acid Binding Protein), GLUT4 (Glucose Transporter 4) and HKII (Hexokinase II) are increased, characterizing a greater uptake of free fatty acids, glucose and phosphorylation of this glucose for further metabolization by muscle tissue, respectively [68]. The increased energy expenditure and the oxidation of lipids imposed by exercise, fasting or not, in obese individuals are able to improve the relationship between mobilization and lipid oxidation. A considerable proportion of the oxidized fatty acids during aerobic exercise of low-to-moderate intensity comes from intramuscular triglycerides, which justifies the use of these exercise protocols in order to improve the insulin sensitivity of its practitioners [36]. Although the present work has not quantified the oxidation of fatty acids per se, it seems that an increased uptake of substrates culminates in the respective increase in their oxidation.

Finally, it must be emphasized that the combination of these two metabolic challenges resulted in an important reduction of blood lipids (triglycerides and total cholesterol). This is of particular interest for the population with obesity and diabetes, since these lipids are known inducers/markers of the development of cardiovascular complications [69].

## 5. Conclusions

We conclude that exercise in the fasted state induces additional positive metabolic changes in fat metabolism that last for up to 12 h after the exercise bout. The reduction of blood triglycerides and total cholesterol (both markers of cardiovascular risk) observed when the two metabolic challenges are associated indicates that this intervention may be particularly important for obese and diabetic subjects. Although no interaction between exercise and fasting was seen, for 12 h after the intervention, in the immunocontent of HSP70 and SIRT1, metabolic differences were found in glycogen and lipid metabolism. Despite the lack of visible interactions between exercise and fasting on HSR and SIRT1, the potential importance of exercise in managing comorbidities of inflammatory origin is evident. In addition, the immunocontent of these proteins might have displayed a different pattern if the exercise recovery intervals were different. It is important to highlight the main limitations of this study: the absence of a non-obese control group and the fact that exercise intensity was not directly measured. Finally, in terms of human health, it is difficult to extrapolate our findings in rats to the human obese population. However, the induced metabolic changes (especially with regard to lower triglycerides and total cholesterol) and the fact that they can last for up to 12 h after the exercise bout are promising findings that need to be investigated in humans.

## Supplementary Materials

The following are available online at https://www.mdpi.com/article/10.3390/ijerph18147543/s1, Figure S1: Blood lactate concentration in fasting (A) and fed (B) conditions of the rats before and after the exercise protocol., Table S1: Effects of fasting exercise on plasma biochemical profile, glycogen, triglycerides storage, and muscle immunocontent of HSP70 and SIRT1.
