# Supplementary material for: Metabolic and Molecular Subacute Effects of a Single Moderate-Intensity Exercise Bout, Performed in the Fasted State, in Obese Male Rats
Source: Int J Environ Res Public Health. 2021 Jul 15;18(14):7543. doi: 10.3390/ijerph18147543 (PMC8307452; doi:10.3390/ijerph18147543)
Supplement: Supplementary file 1 [file ijerph-18-07543-s001.zip › ijerph-1231757-supplementary.pdf]

Supplemental Material

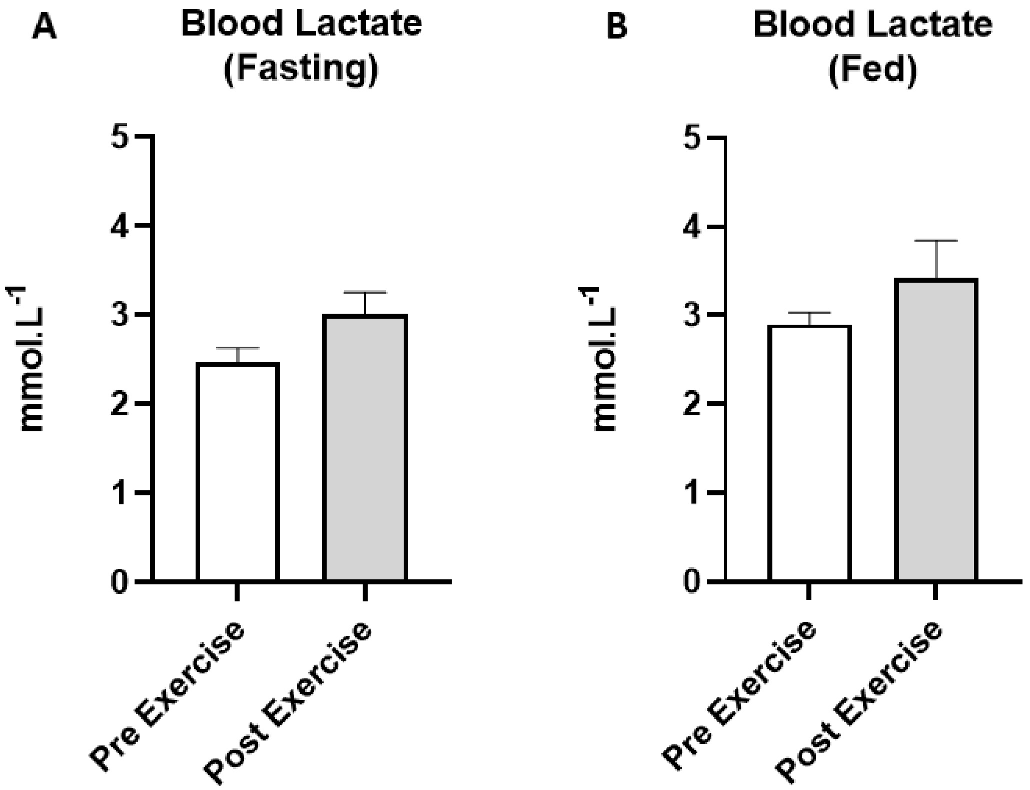

Figure S1. Blood lactate concentration in fasting (**A**) and fed (**B**) conditions of the rats before and after the exercise protocol. Data was analyzed by repeated *t* test. Results were considered different when $p < 0.05$.

Table S1. Results were expressed as mean ± standard error or median (25–75 percentiles). Data were analyzed by two-way ANOVA (Bonferroni post hoc), or Kruskal-Wallis. Results were considered statistically different when $p < 0.05$. *p*1: statistical index for exercise (rest or exercise). *p*2: statisticalindex for fasting (fed or fasted). *p*3: statistical index for the interaction between exercise and fasting. Letters represent significant differences between each experimental group. Bold: significant differences.

Effects of fasting exercise on plasma biochemical profile, glycogen, lactate, triglycerides storage and muscle immunocontent of HSP70 and SIRT-1

	Rest		Exercise				
	Fed (n=8)	Fasting (n=8)	Fed (n=7)	Fasting (n=7)	*p1*	*p2*	*p3*
Serum Glucose ($mg.dL^{-1}$)	91.3 ± 8.53	90.9 ± 11.3	91.2 ± 10.7	79.8 ± 9.03	0.15	0.12	0.15
Serum Total Proteins ($g.dL^{-1}$)	12.5 (11.8 - 12.7) **a**	13.4 (11.7 - 14.2) **a**	12.2 (11.7 - 12.9) **a**	10.9 (10.7 - 11.6) **b**	-	-	**0.01**
Serum Lactate ($mmol.L^{-1}$)	8.27 ± 1.38	8.53 ± 1.29	7.91 ± 1.51	7.76 ± 0.81	0.25	0.89	0.67
Serum Total Cholesterol ($mg.dL^{-1}$)	83.8 ± 17.8 **a**	104.4 ± 36.4 **a**	86.4 ± 23.7 **a**	46.9 ± 7.74 **b**	**0.01**	0.31	**0.01**
Serum Triglycerides ($mg.dL^{-1}$)	193.7 ± 77.6	248.7 ± 107.2	173.7 ± 89.6	69.9 ± 11.5	**0.01**	0.43	**0.02**
Gastrocnemius Glycogen (mg.g of $tissue^{-1}$)	0.37 (0.21 - 2.34)	0.27 (0.19 - 0.68)	0.43 (0.34 - 2.73)	0.27 (0.16 - 0.36)	-	-	0.23
Soleus Glycogen (mg.g of $tissue^{-1}$)	1.41 ± 0.69	0.40 ± 0.18	1.36 ± 0.76	0.58 ± 0.38	0.77	**0.01**	0.58
Hepatic Glycogen (mg.g of $tissue^{-1}$)	73.4 (60.6 - 80.2) **a**	57.3 (38.3 - 77.3) **a,b**	29.3 (24.1 - 62.3) **b**	47.5 (34.2 - 54.4) **a,b**	-	-	**0.03**
Heart Glycogen (mg.g of $tissue^{-1}$)	0.04 ± 0.02	0.04 ± 0.03	0.03 ± 0.02	0.04 ± 0.01	0.96	0.35	0.39
Gastrocnemius Lactate (mg.g of $tissue^{-1}$)	4.16 (1.33 - 6.42)	3.28 (0.84 - 5.41)	1.29 (0.87 - 4.65)	5.54 (2.74 - 6.29)	-	-	0.29
Soleus Lactate (mg.g of $tissue^{-1}$)	2.50 ± 0.76	2.00 ± 0.79	2.53 ± 1.52	2.65 ± 0.35	0.36	0.60	0.39
Hepatic Lactate (mg.g of $tissue^{-1}$)	0.94 ± 0.23	1.13 ± 0.31	1.22 ± 0.33	1.13 ± 0.56	0.30	0.71	0.31
Gastrocnemius Triglycerides (mg.g of $tissue^{-1}$)	2.38 ± 0.84	2.37 ± 0.52	2.78 ± 0.79	2.61 ± 0.56	0.27	0.77	0.78
Soleus Triglycerides (mg.g of $tissue^{-1}$)	2.93 (2.60 - 3.56) **a**	2.34 (2.20 - 2.94) **b**	2.56 (2.35 - 2.72) **a,b**	2.42 (2.01 - 3.01) **b**	-	-	**0.04**
Hepatic Triglycerides (mg.g of $tissue^{-1}$)	14.7 ± 3.29	13.2 ± 2.33	13.2 ± 4.65	14.0 ± 4.15	0.83	0.79	0.41
Heart Triglycerides (mg.g of $tissue^{-1}$)	4.67 ± 0.38 **a**	5.25 ± 0.51 **b**	5.46 ± 0.62 **b**	4.52 ± 0.44 **a**	0.88	0.33	**0.00**
Gastrocnemius HSP70 (AU)	0.30 ± 0.09	0.30 ± 0.09	0.42 ± 0.10	0.48 ± 0.17	**0.00**	0.56	0.48
Soleus HSP70 (AU)	0.40 ± 0.06	0.29 ± 0.05	0.52 ± 0.04	0.49 ± 0.16	**0.00**	0.05	0.25
Gastrocnemius SIRT-1 (AU)	0.25 ± 0.13	0.28 ± 0.11	0.38 ± 0.04	0.48 ± 0.27	**0.01**	0.26	0.52
Soleus SIRT-1 (AU)	0.38 ± 0.07	0.28 ± 0.13	0.40 ± 0.09	0.48 ± 0.19	**0.02**	0.87	0.05
